# Balance between sodium and calcium currents underlying chronic atrial fibrillation termination: An *in silico* intersubject variability study

**DOI:** 10.1016/j.hrthm.2016.08.028

**Published:** 2016-12

**Authors:** Alejandro Liberos, Alfonso Bueno-Orovio, Miguel Rodrigo, Ursula Ravens, Ismael Hernandez-Romero, Francisco Fernandez-Aviles, Maria S. Guillem, Blanca Rodriguez, Andreu M. Climent

**Affiliations:** * ITACA, Universitat Politècnica de València, València, Spain; † Cardiology Department, Hospital General Universitario Gregorio Marañón, Instituto de Investigación Sanitaria Gregorio Marañón, Madrid, Spain; ‡ Department of Computer Science, University of Oxford, Oxford, United Kingdom; § Department of Pharmacology and Toxicology, Technical University Dresden, Dresden, Germany; ¶ Department of Signal Theory and Communications, Rey Juan Carlos University, Fuenlabrada, Madrid, Spain

**Keywords:** Atrial fibrillation, Ionic currents, Rotor dynamics, Calcium current, Mathematical modeling

## Abstract

**Background:**

Atrial remodeling as a result of long-standing persistent atrial fibrillation (AF) induces substrate modifications that lead to different perpetuation mechanisms than in paroxysmal AF and a reduction in the efficacy of antiarrhythmic treatments.

**Objective:**

The purpose of this study was to identify the ionic current modifications that could destabilize reentries during chronic AF and serve to personalize antiarrhythmic strategies.

**Methods:**

A population of 173 mathematical models of remodeled human atrial tissue with realistic intersubject variability was developed based on action potential recordings of 149 patients diagnosed with AF. The relationship of each ionic current with AF maintenance and the dynamics of functional reentries (rotor meandering, dominant frequency) were evaluated by means of 3-dimensional simulations.

**Results:**

Self-sustained reentries were maintained in 126 (73%) of the simulations. AF perpetuation was associated with higher expressions of I_Na_ and I_CaL_ (*P* <.01), with no significant differences in the remaining currents. I_CaL_ blockade promoted AF extinction in 30% of these 126 models. The mechanism of AF termination was related with collisions between rotors because of an increase in rotor meandering (1.71 ± 2.01cm^2^) and presented an increased efficacy in models with a depressed I_Na_ (*P* <.01).

**Conclusion:**

Mathematical simulations based on a population of models representing intersubject variability allow the identification of ionic mechanisms underlying rotor dynamics and the definition of new personalized pharmacologic strategies. Our results suggest that the underlying mechanism of the diverging success of I_CaL_ block as an antiarrhythmic strategy is dependent on the basal availability of sodium and calcium ion channel conductivities.

## Introduction

Pharmacologic treatment of atrial fibrillation (AF) has modest efficacy in terminating the arrhythmia and sustaining sinus rhythm in patients with long-standing persistent AF.^1^, ^2^ One of the explanations for this lack of success in chronic AF patients is the remodeling process of atrial tissue. Prolonged periods of AF result in changes in the characteristics of AF drivers (e.g., dominant frequency [DF], rotor meandering, wavefront curvature) and promote AF maintenance.^3^ Understanding the ionic mechanisms that govern AF drivers will allow the development of more effective antiarrhythmic drug treatments in remodeled substrates. However, the remodeling process and its effects on the interaction between ion channel currents depend on the underlying clinical scenario and genetics of each patient,^4^, ^5^ which may result in perpetuation mechanisms that differ among patients.

Pharmacologic treatments have traditionally attempted to prolong the action potential duration (APD) and refractory period of cells, resulting in an increase of wavelength. However, this strategy has limited efficacy for AF termination and sinus rhythm maintenance.^1^ Another potential strategy, based on most recent knowledge about perpetuation of AF by rotors, is to focus on destabilizing rotor cores. An increase in rotor core movement may promote its extinction by collision with other wavefronts or anatomic obstacles^3^ and thus appears to be an attractive target for antiarrhythmic drugs. Therefore, an in-depth understanding of the ionic mechanisms that govern self-sustained reentries under remodeled conditions is needed. The voltage-dependent sodium current I_Na_ probably is the main ionic current governing wavefront propagation properties during sinus rhythm and reentrant activity. Blockade of this current results in deceleration of reentrant activity and increase in reentry meandering, which facilitates termination of the arrhythmia.^6^ However, I_Na_ block can increase vulnerability to ventricular fibrillation caused by decreased conduction velocity.^2^ In addition, the role of the l-type calcium current I_CaL_ in rotor dynamics remains controversial. It has been observed that I_CaL_ blockade can result in acceleration of fibrillation activity,^7^ consistent with APD shortening,^8^ and in a reduction of fibrillation frequency.^9^, ^10^ The specific mechanisms for these discrepancies remain unclear and can be related to the role of I_CaL_ in terms of propagation.^11^ Our hypothesis is that the effect of I_CaL_ block on atrial rotor dynamics is modulated by the strength of I_Na_ in the specific tissue preparation. Consequently, the specific mechanisms that govern functional rotors may change between patients depending on the relative expression of sodium and calcium currents, opening new venues for personalized pharmacologic strategies to terminate the arrhythmia.

In order to validate this hypothesis, a population of 173 mathematical models capturing variability in experimental measurements from 149 AF patients was used to evaluate the role of each ionic current in the dynamics of functional reentries. The effect or I_CaL_ blockade on reentrant biomarkers and its efficacy for AF extinction by destabilizing the core of rotors were evaluated. Intersubject variability allows identification of the mechanisms that produce diverging effects on AF characteristics by the same antiarrhythmic treatment depending on the basal expression of ion channels.

## Methods

### Experimental dataset and biomarkers

**Figure 1:**
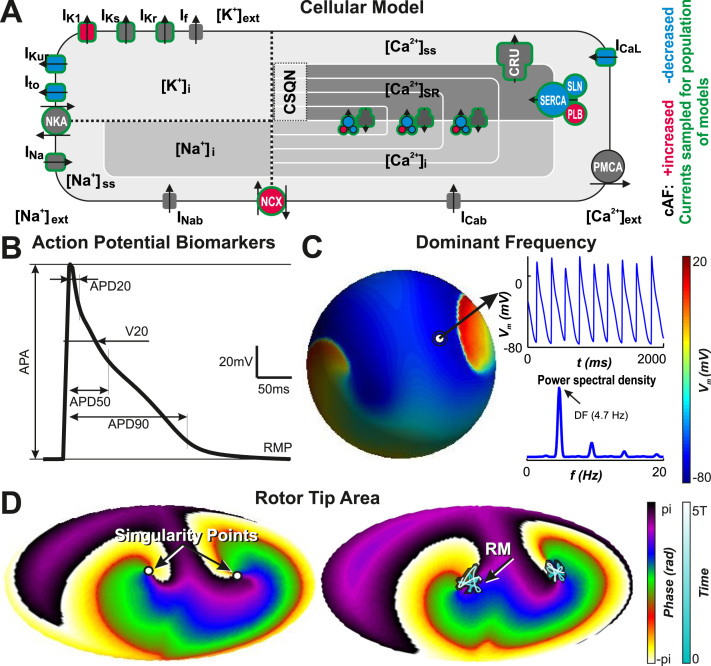
Parameters and biomarkers evaluated in the study. **A:** Koivumaki model. Atrial fibrillation modifications are depicted in *red* and *blue.*^8^ Currents sampled to obtain the population of models are shown in *green.***B:** Action potential biomarkers: APD_20_, APD_50_, APD_90_, action potential amplitude (APA), resting membrane potential (RMP), and V_20_. **C:** Membrane voltage in sphere simulations according to color scale. *Insets* show transmembrane voltage and power spectral density for a given node, illustrating the dominant frequency (DF). **D:** Phase maps in Aitoff projection of the sphere. **Left:** Detection of the rotor core. **Right:** Meandering of the core according to color scale. APD = action potential duration.

Action potential (AP) recordings in atrial trabeculae samples (n = 215) of right atrial appendages from 149 patients diagnosed with chronic AF were available.^2^, ^5^ The following AP biomarkers were quantified at 1 Hz (Figure 1): APD at 20%, 50%, and 90% of repolarization (APD_20_, APD_50_, APD_90_, respectively), action potential amplitude (APA), resting membrane potential, and AP plateau potential at 20% of APD_90_ (V_20_).

In addition to AP recordings at 1 Hz, a subset of the preparations (n = 9) was used to characterize rate dependency in human atrial APs by quantifying rate-sensitive biomarkers (APD_50ratio_ and APD_90ratio_) as the ratio between APD at each frequency (2, 3, and 4 Hz) and at 1 Hz for APD_50_ and APD_90_.

### Population of models

An experimentally calibrated population of human AF models was generated from the experimental data described earlier, the Latin hypercube sampling methodology described in by Britton et al,^12^and the baseline “AF model” by Koivumaki et al.^8^ Therefore, ionic conductances highlighted in green (Figure 1A) took random-sampled values between –100% to +200% of their original value.

The 173 models of the initial population of 16,384 mathematical models that satisfied at all pacing frequencies the physiologic range of biomarkers (as identified in the experimental recordings) constituted the AF population (see Online Supplementary Material).

### Role of ionic currents on AF reentrant mechanisms

The impact of intersubject variability in ionic properties in AF-related rotor dynamics was evaluated using 173 computer simulations (one for each of the models in the AF population) conducted in a 3-dimensional (3D) spherical atrial tissue model with membrane kinetics. Fibrillation or rotor activity was characterized using DF and the area of rotor meandering (RM) (Figure 1 and Online Supplementary Material).

In order to identify the main ion channel conductivities involved in the perpetuation of reentries, the 11 modified conductivities were compared between the groups of models with sustained vs unsustained reentries. Relations between modified ionic currents, 1-Hz AP biomarkers, and AF reentrant characteristics (DF, RM) also were analyzed.

### Effects I_CaL_ blockade in the population of models

Simulations described in the previous section were analyzed during basal conditions and after a 50% reduction in calcium conductivity (g_CaL_). The 3D models in which I_CaL_ reduction terminated the arrhythmia were compared with those that maintained reentrant activity. The effect of I_CaL_ block in both DF and RM and its relationship with the modified ionic currents and 1-Hz biomarkers were evaluated.

### Statistical analysis

The Mann–Whitney *U* test, which is robust against nongaussian distributions, was used to evaluate statistical significance between variables (*P* <.01).

To evaluate the role of each of the modified currents on rotor dynamics (RM, DF), partial correlation coefficients (PCr) were used.^12^

## Results

### Role of ionic currents on reentrant AF mechanisms

The simulation of 173 different physiologic atrial tissue models allowed us to identify (1) differences between models that do and do not allow AF maintenance and (2) the role of each ion current conductance on AF characteristics (i.e., DF and RM).

**Figure 2:**
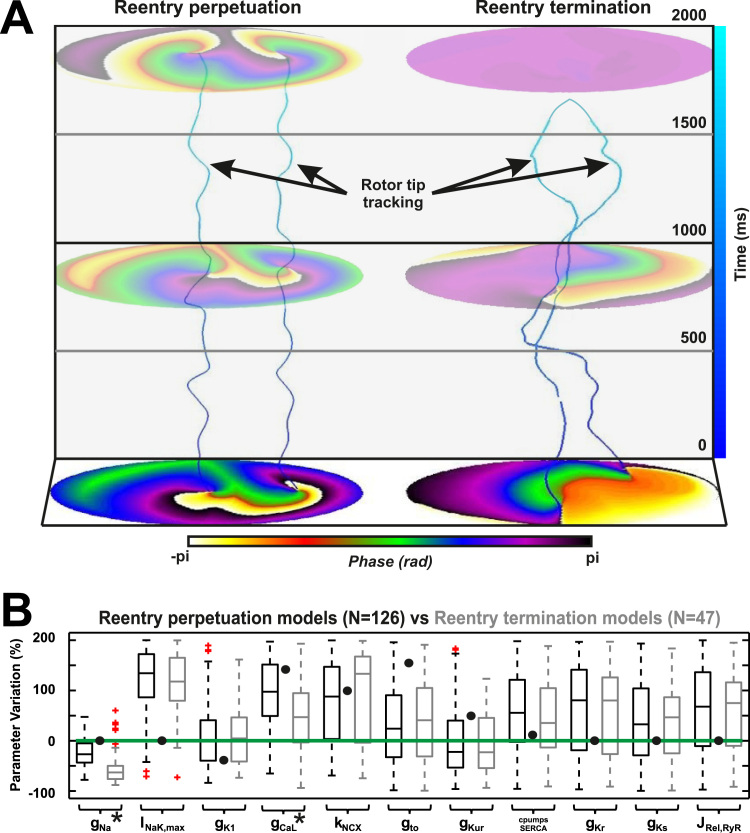
End of functional reentry and underlying ionic parameters. **A:** Phase maps and filaments tracking phase singularities in time. **Left:** Spatially stable rotors maintaining reentrant activity. **Right:** Larger rotor meandering resulting in reentry annihilation by the collision of rotor cores. **B:** Box plots of ionic current parameters in models maintaining *(black)* and not maintaining *(gray)* functional reentries. *P <.01. *Green line* and *black dots* indicate baseline atrial fibrillation and sinus rhythm models, respectively.

Arrhythmias were self-terminated in 47 (27%) of the models during the first seconds of simulated reentrant activity. The temporal evolution of phase maps for representative examples in each subgroup is shown in Figure 2. Note that in the example on the left, reentries were stable during the entire simulation. However, in the example on the right, although reentries start in a similar position, rotor cores drifted up to collision, where the arrhythmia terminated. This mechanism of termination (i.e., collision between rotors due to larger RM) was present in all the 47 simulations in which the arrhythmia was not perpetuated. This result suggests that ion channel currents involved in RM may play a fundamental role in the perpetuation or termination of AF rotors.

A comparison of the distribution of each ionic current parameter between the models in which the fibrillation terminated and those in which it was perpetuated is depicted in Figure 2B. Sodium and calcium conductivities (g_Na_ and g_CaL_) were the only 2 parameters that presented significant differences between both groups, highlighting their relevance as possible antiarrhythmic targets. Interestingly, no significant differences were observed on potassium repolarizing currents, which previously have been proposed as potential antiarrhythmic targets for AF.^1^, ^2^

**Figure 3:**
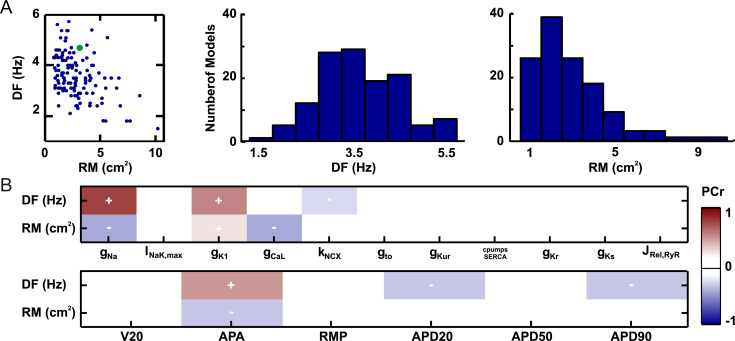
Ionic currents and functional reentries. **A:** Scattergram and histograms of dominant frequency (DF) and rotor meandering (RM), showing the distributions of both biomarkers for the 126 AF models that maintained reentry. *Green dot* represents the baseline model. **B:** Partial correlation coefficients (PCr) between ionic parameters and reentry biomarkers **(top)** and AP biomarkers at 1 Hz **(bottom).** Darker colors represent stronger correlations. APA = action potential amplitude; APD = action potential duration; RMP = resting membrane potential.

Specific AF characteristics of the models in which the arrhythmia was perpetuated are shown in Figure 3. Significant variability can be observed in the distribution of DFs and RMs, which indicates that the developed population of models allows for the simulation of multiple AF scenarios. Because of the intersubject variability introduced by this database, the relationship between ion channel parameters and AF biomarkers can be identified. Despite the low direct correlation observed between DF and RM (R^2^ = –.44), the scatterplot indicates that high DFs are most often related with low RMs, whereas low DFs can be found with any RM value. This result takes on additional relevance when related with the partial correlation analysis between AF biomarkers and ion channels properties (Figure 3B). Sodium conductance plays a main role in both parameters. Although an increase of g_Na_ is correlated with an increase of DFs, it also is related to a reduction of RM. However, the lack of a strong correlation between DF and RM indicates that the effect of g_Na_ in both AF biomarkers is significantly affected by other conductivities. Specifically, our results suggest that, in addition to g_Na_, DF is mainly governed by g_K1_, whereas RM is inversely related to g_CaL_. None of the remaining studied parameters of the model presented a significant partial correlation with AF characteristics.

Regarding the relationship between AF characteristics and AP biomarkers, our results indicate that APA is the only biomarker related with both RM and DF. The large dependence of reentrant biomarkers with g_Na_ is in accordance with the dependency of APA with g_Na_ (see Online Supplemental Figure 2). In addition to APA, both APD_20_ and AP_90_ presented minor correlations with DF, but we found no strong correlation between any biomarker and RM.

### I_CaL_ blockade effects in the population of models

The findings that RM plays a relevant role in the termination of AF as a result of rotor collisions, and that RM is mainly governed by g_Na_ and g_CaL_ (PCr = –0.36 and –0.42), raise questions regarding the efficacy of I_CaL_ blockers depending on the relative expression of other ion channel currents.

**Figure 4:**
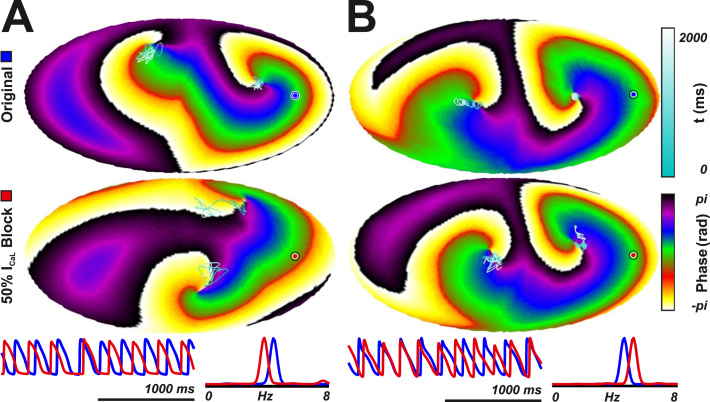
Effects of calcium current block on dominant frequency (DF) and rotor meandering (RM) in 2 representative models of DF decrease **(A)** and increase **(B).** Phase maps are depicted for 1 time instant, together with RM in *light blue.* Transmembrane voltage and power spectral density **(bottom)** are shown for selected nodes under baseline conditions *(blue)* and 50% I_CaL_ reduction *(red).*

Specifically, effects of a 50% reduction of g_CaL_ on AF characteristics were evaluated in the 126 models that maintained fibrillation during basal conditions. The juxtaposed effect of this reduction on AF characteristics in 2 examples is depicted in Figure 4. Note that the reduction of g_CaL_ produced a significant increase in RM (from 1.59 to 3.49 cm^2^) and a decrease in DF (4.4 vs. 3.8 Hz) (Figure 4A), whereas the reduction of g_CaL_ resulted in an increase in DF (4.6 vs. 5.1 Hz) (Figure 4B).

**Figure 5:**
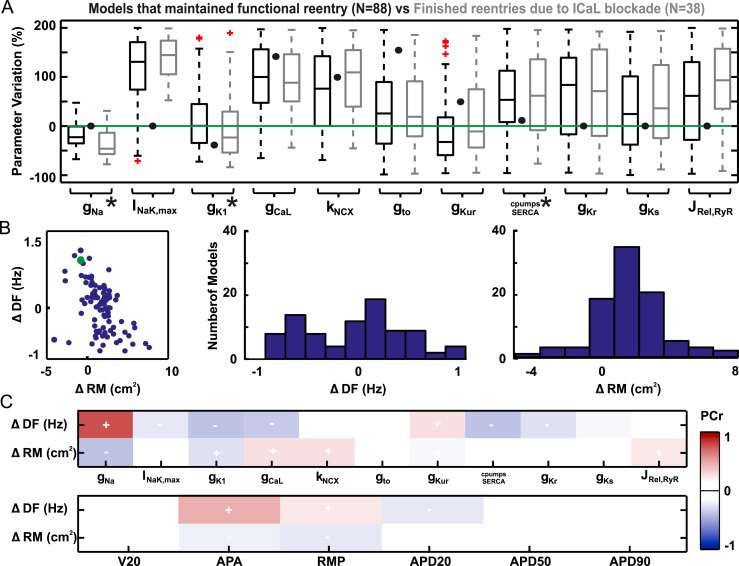
I_CaL_ blockade in the whole population of models. **A:** Box plots of ionic current parameters in models exhibiting unsuccessful *(black)* and successful *(gray)* reentry termination by I_CaL_ block. **B:** Scattergram and histograms of dominant frequency (DF) and rotor meandering (RM) variations in models in which reentry was perpetuated. **C:** Partial correlation coefficients (PCr) between variations in reentry properties and ionic currents **(top)** and action potential biomarkers at 1 Hz **(bottom).** APA = action potential amplitude; APD = action potential duration; RMP = resting membrane potential.

The reduction of g_CaL_ also had different degrees of success on AF termination depending on the specific characteristics of each model. After 7 seconds of simulation under the effects of g_CaL_ block, 38 of the 126 reentrant processes (30%) terminated. The differences between ion channel properties in the cases of abolished and persisting arrhythmias are shown in Figure 5. Models in which I_CaL_ block resulted in reentry termination presented lower expressions of I_Na_ and I_K1_ (Figure 5A). This is suggestive of the potential implication of patient-specific ratios between g_CaL_, g_Na_, and g_K1_ on the antiarrhythmic effects of I_CaL_ blockers.

The important role of this balance between ion currents in the specific mechanisms by which calcium block terminates AF is shown in Figure 5B. The reduction in g_CaL_ resulted in an average increase in RM. However, this effect was not uniform in the entire population, in some cases resulting in a reduction of RM and therefore stabilization of the arrhythmia. Regarding the modifications on DF, no clear trends were observed. In addition, as during basal conditions, a weak inverse relationship between DF and RM was observed (R^2^ = –0.48). The partial correlation between changes in DF and RM and each of the model parameters can shed some light on the characteristics of calcium blockers responders and nonresponders. As shown in Figure 5C, g_Na_ was the main parameter correlated with both DF and RM changes. According to these results, the inverse correlation between g_Na_ and the change in RM indicates that calcium blockers produced an increase in RM mainly in models in which the basal conductance of I_Na_ was low. This result confirmed that the antiarrhythmic effect of calcium blockers could be subjected to the degree of expression of the sodium current in each patient.

In the case of DF, a more complex behavior is observed. The direct correlation between changes in DF and g_Na_ is suggestive of an increase in DF due to calcium block in models with higher sodium channel conductance. However, the inverse correlation between the change in DF and g_K1_ points to a reduction of DF in models in which g_K1_ was high, as the strong repolarizing effect of I_K1_ is not compensated by I_CaL_. This multifactorial response to calcium block may explain the controversial correlation between calcium blockers and modifications on DFs reported in the literature.^7^, ^9^, ^10^

## Discussion

### Major findings

In this study, a population of 173 mathematical models that mimics the intersubject variability of 149 AF patients including rate-dependent response was used to evaluate the role of each ionic current on AF reentry perpetuation mechanisms by means of 3D spherical models. The fundamental and interdependent role of I_Na_, I_CaL_ and I_K1_ currents on AF dynamics has been shown. This physiologic variability in ionic current densities may independently explain the observed disparity of antiarrhythmic drug effects, which can be related to the expression of other currents untargeted by a compound. As shown here, this may be the case of calcium blockers, whose antiarrhythmic effect is dependent on sodium current density.

### AF population of models

Mathematical models have been used to evaluate hypotheses of AF perpetuation mechanisms and drug response since the late 1950s. During the last decades, as result of the increase in computing power together with more extended research in cardiac ionic currents by patch-clamp experiments, more sophisticated electrophysiologic models have been developed. These ionic current models have been used to evaluate the effect of antiarrhythmic treatments on both AP and rotors dynamics.^8^, ^13^ However, most of these *in silico* studies have made use of a single set of parameters fitted to an average of many experiments. Although this approach has allowed the reproduction and better understanding of AF mechanisms, it hides the variability between patients observed in clinical practice. This variability may explain the contradictory responses to pharmacologic treatments reported in the literature. New cardiac simulation platforms such as those used in this work^14^ allow performance of simulations of populations of models, accounting for the variability observed across subjects. Furthermore, this is the first study in which a population of models has been used to determine the ionic currents linked to arrhythmia perpetuation in a 3D model of AF.

AP markers from experimental recordings allowed us to select ionic current combinations that result in APs covering the observed experimental range. As in previous studies,^5^, ^12^ variability in ionic parameters reproduces AP variability in agreement with the biomarkers obtained from the recorded preparations. In order to account for the rate dependence of atrial electrophysiology, we included restrictions in our population reproducing the observed trends. Of note, the introduction of these rate-dependence bounds resulted in the rejection of models with unrealistic calcium transients and highlighted the relevance of I_NaK_ in rate dependence, in accordance with previous reports.^15^ To the best of our knowledge, this is the first time that the APD rate dependence relative to basal APD at 1 Hz has been used to select the most physiologically relevant AF models from among a wider population (see Online Supplemental Figure 1), therefore allowing a more realistic response of the models under fast activation rates such as those observed during AF.

### Strategies for the development of new pharmacologic therapies and reentrant biomarkers

Clinical and research studies on AF have demonstrated the importance of rotors on AF maintenance.^6^, ^10^, ^16^ Overexpression of repolarization currents, such as I_K1_ or I_KACh_, resulted in rotor acceleration as a consequence of APD shortening,^1^, ^17^ whereas I_K1_ block has been shown to reduce DF.^18^ However, studies on pharmacologic block of these currents have reported a modest effect on AF termination and sinus rhythm maintenance.^1^ Our simulations in the population of models show a significant correlation of I_K1_ with DF, and no relationship with I_K1_, I_Kur_, I_Kr_, or I_Ks_ in terms of AF maintenance (Figure 2B).

These results suggest that AF maintenance, as previously hypothesized,^6^, ^9^, ^10^ may be more strongly related to the depolarizing currents I_Na_ and I_CaL,_ which increase RM when decreased while yielding a reduction on APD_90_. Reduced I_Na_ or I_CaL_ availability results in decreased excitability, forcing the rotor core to drift. This increased RM may facilitate annihilation of rotors due to collision, as found in the present study and others.^6^, ^9^, ^10^

According to these results, AF termination might be approached by pharmacologic reduction of I_CaL_. However, the role of I_CaL_ as an antiarrhythmic drug is controversial, as both acceleration^7^ and deceleration^9^, ^10^ of fibrillatory behavior have been reported. This controversy has been also previously discussed in terms of the lack of specificity of some drugs, such as verapamil, which also blocks I_Kr_.^19^ In contrast, simulations allowed us to specifically block I_CaL_. Yet, a significant number of models could not maintain the arrhythmia even though a significant correlation between I_CaL_ block and DF was not found. This could be explained by the simultaneous effect of I_CaL_ on APD and RM^8^ (see Online Supplementary Material) because it shortens APD (which would tend to increase DF) but at the same time increases RM (which, in turn, tends to decrease DF). The balance between these 2 opposing effects has been shown to be multifactorial but strongly related to I_Na_ availability. Therefore, I_CaL_ may be effective for AF termination in cases of low availability of I_Na_.

According to the results of our study, the antiarrhythmic effect of calcium channel blockers may be more prominent in some patients than others, depending on their specific balance of ion channel currents, particularly I_Na_.

These results allow us to suggest personalized antiarrhythmic treatments based on the patient’s expression of different ion channels. For example, low expression of I_Na_ could indicate a positive responder to I_CaL_ block antiarrhythmic treatment, whereas calcium blockers may be ineffective in patients with a high expression of I_Na_. Thus, protein expression techniques might help in the stratification of patients depending on I_Na_ levels in atrial tissue to select appropriate candidates for I_CaL_ block therapy.

Populations of models highlighted differences between responders and nonresponders to treatments (I_CaL_ block in this case). These analyses will be helpful in the development of new antiarrhythmic strategies and in the selection of responder patients to both established and novel treatments, such as I_Na_ block or multichannel drugs. Furthermore, our results reinforce the development of drugs that partially block both I_CaL_ and I_Na_.

### Study limitations

Mathematical models are partial representations of real objects, so their results can be conditioned by gaps in knowledge. Additional repolarizing currents, such as small-conductance calcium-activated potassium channels, are increasingly recognized as contributors to AF remodeling.^20^ However, the inclusion of such channels should not alter the main findings of this study, based on the balance between atrial depolarizing currents and tested here against a wide range of possible repolarization reserves. Whereas extrapolation of results requires caution and future validation against refined models of human electrophysiology, the introduction of intersubject variability approximates *in silico* experiments to more realistic clinical scenarios.

Because of technical limitations, tissue samples were obtained from the right atrial appendage and thus may not be representative of the entire atria. The left atrium has been reported as more frequently involved in AF maintenance in paroxysmal AF^16^, ^17^, whereas right atrial tissue of those patients may harbor reentries with lower DFs. This may be one of the mechanisms of our reported frequencies, which are lower than those typically found in AF patients.

In addition, structural disarrangements (e.g., fibrosis or decreased tissue coupling) play a relevant effect in excitability and rotor maintenance.^11^, ^21^ Channel kinetics also have an important role in the initiation and maintenance of atrial arrhythmias, as in the case of late I_Na_.^22^ Further studies investigating the relationship between these parameters and the properties of reentrant activity are needed.

Finally, the morphology of simulated atrial tissue does not reproduce the complex atrial anatomy in humans. Nevertheless, introduction of anatomic heterogeneities would most likely increase the incidence of collisions and annihilation of wavefronts under increased RM.

## Conclusion

Experimentally calibrated populations of models are presented as a useful tool for understanding the ionic mechanisms related to rotor dynamics and defining new pharmacologic targets for AF. This study showed that the same pharmacologic treatment can produce different effects on AF dynamics for cells modeled under the same variability found in human experimental data. By using this computational framework, our results suggest that I_CaL_ block can be an efficient antiarrhythmic treatment in AF patients with depressed I_Na_ current. This methodology will be very helpful in the selection of responder patients and the development of new antiarrhythmic treatments, such as I_Na_ block or drugs that affect multiple channels.

## Footnotes

Appendix Supplementary data associated with this article can be found in the online version at 10.1016/j.hrthm.2016.08.028.
