# Supplementary material for: Balance between sodium and calcium currents underlying chronic atrial fibrillation termination: An in silico intersubject variability study
Source: Heart Rhythm. 2016 Dec;13(12):2358–65. doi: 10.1016/j.hrthm.2016.08.028 (PMC5221730; doi:10.1016/j.hrthm.2016.08.028)
Supplement: Supplementary file 1 — Supplementary material [file mmc1.pdf]

Supplementary Material

Title:

Balance between Sodium and Calcium Currents Underlying Chronic Atrial Fibrillation Termination: An In Silico Inter–subject Variability Study

Authors:

Alejandro Liberos, PhD[a,b], Alfonso Bueno-Orovio, PhD[c], Miguel Rodrigo, MS[a], Ursula Ravens, MD[c], Ismael Hernandez-Romero, MS[b,e], Francisco Fernandez-Aviles, MD PhD[b], Maria S Guillem, PhD[a],Blanca Rodriguez, PhD[c]*, Andreu M Climent, PhD[b]*

*Equally contributing senior authors.

[a]ITACA, Universitat Politécnica de València, València, Spain. Camí de Vera s/n 46022.

[b]Cardiology Department, Hospital General Universitario Gregorio Marañón, Instituto de Investigación Sanitaria Gregorio Marañón, Madrid, Spain. C/Dr Esquerdo 46 28007.

[c]Department of Computer Science, University of Oxford, Oxford, UK. Parks Rd OX1 3QD.

[d]Department of Pharmacology and Toxicology, Technical University Dresden, Dresden, Germany. Fetscherstr. 74, 01307.

[e]Department of Signal Theory and Communications, Rey Juan Carlos University, Fuenlabrada, Madrid, Spain. Camino del Molino s/n, 28943

Extended Methods

Electrophysiological Cellular Model

The human atrial AP model by Koivumaki et al. was used as the basis for this study, since it presented more stable rotor cores in comparison with other atrial cell models [1]. The electrophysiological remodeling caused by chronic AF was introduced as in Koivumaki et al. [2] to implement a baseline AF model (Figure 1A) by altering: g_{CaL} (–59%), g_{to} (–62%), g_{Kur} (–38%), g_{K1} (+62%), k_{NCX} (+50%), expression of Sarcoplasmic reticulum Ca^{2+} ATPase pump (SERCA, –16%) and the modulating effect of Phospholamban and Sarcopilin in SERCA (+ 18% and -40%).

Population of Models

The Latin Hypercube Sampling method [3,4] was used to generate 16.384 combinations of the currents listed: I_{NaK}, g_{K1}, g_{CaL}, k_{NCX}, g_{to}, g_{Kur}, g_{Kr}, g_{Ks} as well as the intake and release of Ca^{2+} from sarcoplasmic reticulum (cpumps in SERCA and $J_{rel,RyR}$ respectively) from the chronic AF model by Koivumaki [2]. The method allows the generation of a combination of the N=11 parameters by means of sampling the N–dimensional space with a high resolution, efficiently and without bias. It is implemented by stablishing the

inferior and superior and bounds (–100 to +200%) and sampling the range in each parameter in M=16.384 intervals, which results in M^N locations in the N–dimensional space. Then, M of these locations are randomly chosen satisfying that, for each dimension N, each interval is not chosen more than once.

Note that our methodology aims to considering a wide variability in the balance between ionic currents, where the calibration against experimental data (at multiple pacing frequencies, multiple action potential biomarkers) reinforces the physiological plausibility of the selected models. It is defined as an experimentally-calibrated population of models which represents differences with other approaches studying variability as sensitivity-analysis-based methods or cell-specific modelling [5]. The inferior (-100%) and superior (+200%) were selected in order to reach a complete block of the given current, while the superior bound (+200%) were selected according to the strong upregulations and downregulations in channel expression reported in the literature regarding AF remodeling [2]. The 16,384 candidate cellular models were simulated at different pacing frequencies: 1, 2, 3 and 4 Hz with square stimulus of duration 2 ms and amplitude 1250 pA, followed by negative amplitude during the inactive time interval to maintain current conservation in the model, and simulate the effect of neighbouring cells in the tissue. Mathematical simulations were performed on a cardiac simulation GP–GPU platform [6]. APs from period 91 to 100 were stored at sampling frequency of 1 kHz and used to measure the AP biomarkers depicted in Figure 1.

The identification of the subset of models that will be retained in the AF population was carried out in two different steps.

A first calibration filter was applied to select the AF models yielding biomarkers APD90, APD50, APD20, APA, RMP and V20 at 1 Hz within experimental range. In addition, to avoid non-physiological AP upstrokes, the time between the stimulus and AP peak was imposed to be shorter than 20 ms and dV/dt_{max} higher than 20 V/s.

A second calibration filter was applied to reproduce rate dependence in APD of human AF models in the population as in the experimental data. The human AF models yielding APDratios for APD90 and APD50 at 2, 3 and 4Hz within the regression bounds estimated from the experimental recordings were finally accepted (Figure S1B).

Tissue simulations

Briefly, propagation of electrical excitation was simulated using the monodomain equation over a spherical geometry of a 3 cm radius incorporating 163,842 nodes, with an average inter–nodal distance of 283.25 ±

18.42 μm [7], membrane capacitance (50pF) [2], and diffusion coefficient of 0.12 mm^2/ms [8]. The simulations were run in a GP-GPU platform using a combination between the forward Euler and Rush-Larsen scheme as described in [6]. Initiation of reentrant activity in the spherical model was attempted using a cross stimulation protocol. It initiated a figure of eight reentry that resulted in two (or more, in the case of breakthroughs) singularity points. Two seconds of transmembrane voltage maps were analyzed after five seconds of simulation for each AF model to determine whether reentry was initiated and/or sustained. Rotor activity and/or fibrillation were characterized using the dominant frequency (DF) and the area of rotor meandering (RM) (Figure 1). Specifically, DF was computed by using the Welch periodogram (1.25 second Hamming window with a 8192 point Fast Fourier Transform per window and 80% overlap). In order to measure RM, phase maps were computed and singularity points were detected as described in [7]. Only singularity points that accounted for at least 3 consecutive rotations were considered as sustained reentries. In sustained reentry models, RM was quantified for up to 5 consecutive rotations.

Extended Results

Generation of the Population of AF Mathematical Models

Figure S1 illustrates results of the experimental calibration of the population of human AF models as described in Methods. From the initial 16,384 human atrial models, 945 (5.79%) models (depicted in gray) yielded biomarkers in range with biomarkers at 1Hz (light blue square [9]) and constituted the 1Hz population. Only 173 (1.06%) of the models also met the rate dependence constraints (depicted in blue) and constituted the final 'AF population'. Therefore, although a larger number of ion channel combinations reproduced 1Hz experimental recordings for a pacing rate of 1 Hz, only few of them were physiologically realistic in terms of pacing rate dependence. Simulated biomarkers for the human AF models nicely overlap with the experimental biomarkers, covering the experimental range for the biomarkers at 1Hz (Figure S1A) and rate dependency (Figure S1B). Notice that for both experimental and simulated values, the larger the APD at 1 Hz the lower the APDratio, indicating a more pronounced APD shortening as a function of pacing rate and baseline APD.

Interestingly, the original 'AF model' (green point) met most constraints imposed, except for the APD50ratio at 3Hz, although biomarkers tended to be close to the low biomarker limits rather than average behavior. Figure S1C shows APs and calcium transients for AF models through the experimental calibration process. The introduction of rate dependence biomarkers reduced the variability observed in calcium

transients when only APD biomarkers at 1 Hz were taken into account.

The specific effects of each parameter under study in each biomarker, analyzed by partial correlation (PCr) on the physiological population, are presented in Figure S2. Consistent with previous studies, ADP90 is inversely related to g_{K1} and g_{Kur}, and directly related to the conductivities of g_{CaL} and k_{NCX}. An increase in g_{Na} directly mainly affects APA.

Figure S2B shows the distributions of ionic properties for the 173 models of the AF population and those 945 models that reproduced only the 1Hz biomarkers (i.e. 1Hz population).

As a result of accounting for physiological variability, it is of note that most currents inside the 1 Hz and AF populations were not circumscribed to the midpoint of the simulated range (-100% to +200%) and showed median values that often separated from the average AF model. While the median values for g_{K1} and g_{Kur} within the human atrial population matched their baseline values, the median value for g_{Na} was decreased with respect to baseline. The median values for g_{CaL} and k_{NCX} in the population were larger than those in the AF model and closer to those in the sinus rhythm model.

The parameters that differed from 1 Hz and physiological populations to a greater extent were g_{Na}, $I_{NaK,max}$ and g_{Kr} ($p<0.01$). In particular, g_{Na} tended to be lower while $I_{NaK,max}$ and g_{Kr} tended to be larger to fulfill the imposed rate-dependence criteria. This highlights the importance of I_{NaK} in AP rate adaptation, consistent with previous works [10].

References:

1. Wilhelms M, Hettmann H, Maleckar MM, Koivumaki JT, Doessel O, Seemann G. Benchmarking electrophysiological models of human atrial myocytes. Front Physiol 2013; 3:487.

2. Koivumaki JT, Seemann G, Maleckar MM, Tavi P. In Silico Screening of the Key Cellular Remodeling Targets in Chronic Atrial Fibrillation. PLoS Comput Biol 2014; 10:e1003620.

3. Mckay M, Beckman R, Conover W. A comparison of three methods for selecting values of input variables in the analysis of output from a computer code. Technometrics 2000; 42:55-61.

4. Britton OJ, Bueno-Orovio A, Van Ammel K, Lu HR, Towart R, Gallacher DJ, Rodriguez B. Experimentally calibrated population of models predicts and explains intersubject variability in cardiac cellular electrophysiology. Proc Natl Acad Sci USA 2013; 110:E2098-E2105.

5. Muszkiewicz A, Britton OJ, Gemmell PM, Passini E, Sánchez C, Zhou X, Carusi A, Quinn TA, Burrage K, Bueno-Orovio A, Rodriguez B. Variability in cardiac electrophysiology: Using experimentally-calibrated populations of models to move beyond the single virtual physiological human paradigm. Prog Biophys Mol Biol 2015; 120:115-127

6. Garcia-Molla VM, Liberos A, Vidal A, Guillem MS, Millet J, Gonzalez A, Martinez-Zaldivar FJ, Climent AM. Adaptive step ODE algorithms for the 3D simulation of electric heart activity with graphics processing units. Comput Biol Med 2014; 44:15-26.

7. Rodrigo M, Guillem MS, Climent AM, Pedron-Torrecilla J, Liberos A, Millet J, Fernandez-Aviles F, Atienza F, Berenfeld O. Body surface localization of left and right atrial high-frequency rotors in atrial fibrillation patients: A clinical-computational study. Heart Rhythm 2014; 11:1584-1591.

8. Bueno-Orovio A, Kay D, Grau V, Rodriguez B, Burrage K. Fractional diffusion models of cardiac electrical propagation: role of structural heterogeneity in dispersion of repolarization. J R Soc Interface 2014; 11:20140352.

9. Sanchez C, Bueno-Orovio A, Wettwer E, Loose S, Simon J, Ravens U, Pueyo E, Rodriguez B. Inter-Subject Variability in Human Atrial Action Potential in Sinus Rhythm versus Chronic Atrial Fibrillation. PLoS One 2014; 9:e105897.

10. Bueno-Orovio A, Sanchez C, Pueyo E, Rodriguez B. Na/K pump regulation of cardiac repolarization: insights from a systems biology approach. Pflugers Arch - Eur J Physiol 2014; 183-193.

11. Koivumaki JT, Korhonen T, Tavi P. Impact of Sarcoplasmic Reticulum Calcium Release on Calcium Dynamics and Action Potential Morphology in Human Atrial Myocytes: A Computational Study. PLoS Comput Biol 2011; 7:e1001067.

Figure S1. Calibration of the population of models. (A) The values of the biomarkers –V20, APD20, APD50, APD90, RMP and APA– are shown. The green dot represents the baseline AF model; blue and gray dots correspond to the 'AF population' obtained with the calibration including rate dependence constraints (N=173) and those that only reproduced the conditions of biomarkers at 1Hz (N=945), respectively. Orange marks correspond with a random sort of values measured in experimental preparations. Upper and lower bounds for 1 Hz biomarkers are marked in light blue. (B) Rate dependence of APDs and rate dependence bounds. Only the models with a deviation from regression lines –depicted in red– below the maximum deviation observed in experimental measurements were accepted. Note that models that fulfill one but not all criteria may appear as gray dots inside some upper and lower bounds for a given pair of biomarkers. (C) AP and intracellular calcium traces are depicted. Yellow traces correspond to all the simulated models (N=16,384), whilst the rest of color traces correspond to those described above.

Figure S2. Ionic current parameters and correlation with biomarkers. (A) Partial correlation coefficient (PCr) values between ionic parameters and AP biomarkers. PCr values are represented with colors according to the color bar, darker colors represent stronger correlation. (B) Boxplot of ionic current parameters in the population of models. The boxplots in black correspond to the AF population fulfilling the constraints of biomarkers at 1Hz and rate dependence calibration, whereas those in gray correspond to the 1Hz population (*$p<0.01$). The green line represents the baseline AF model values, while black dots represent the sinus rhythm model by Koivumaki [2,11].

170 Figure S1

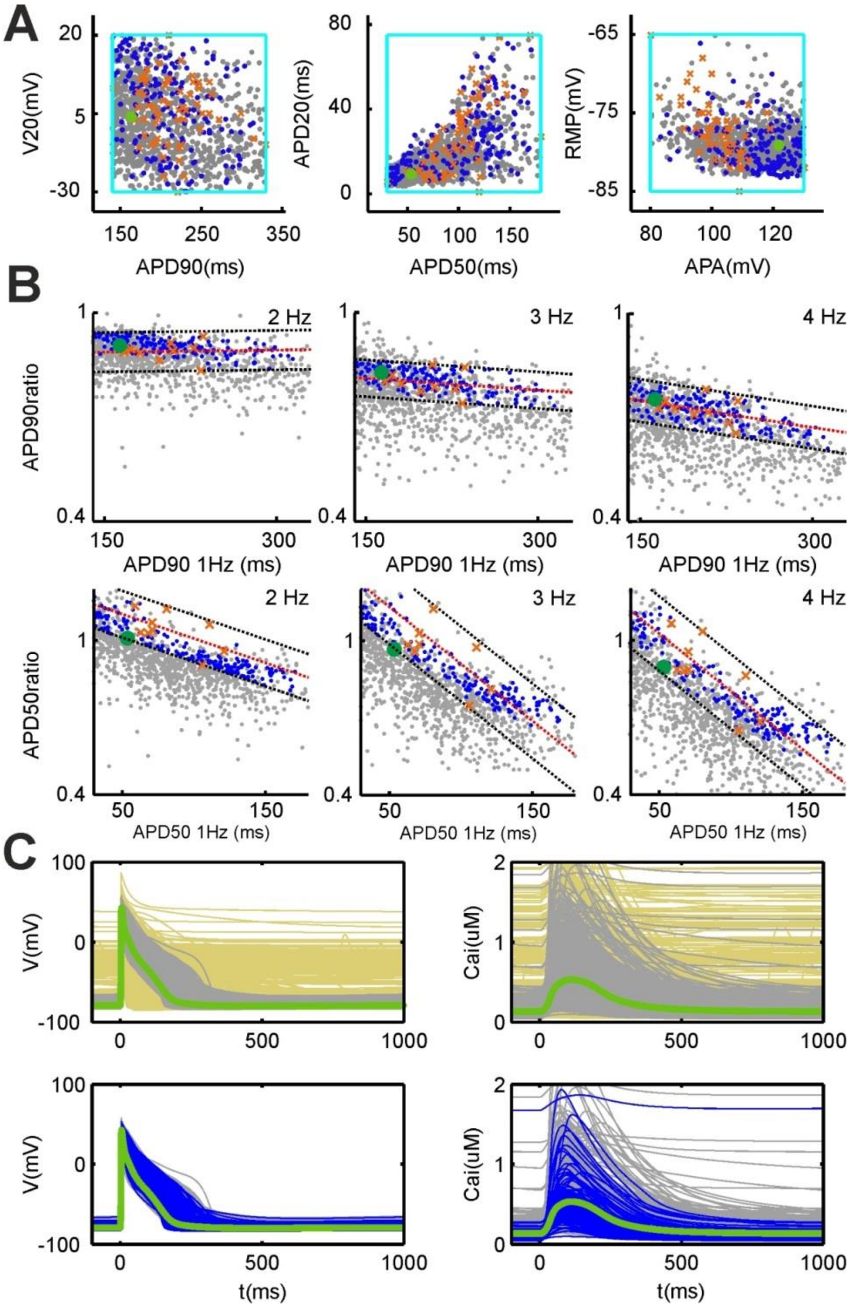

Figure S2

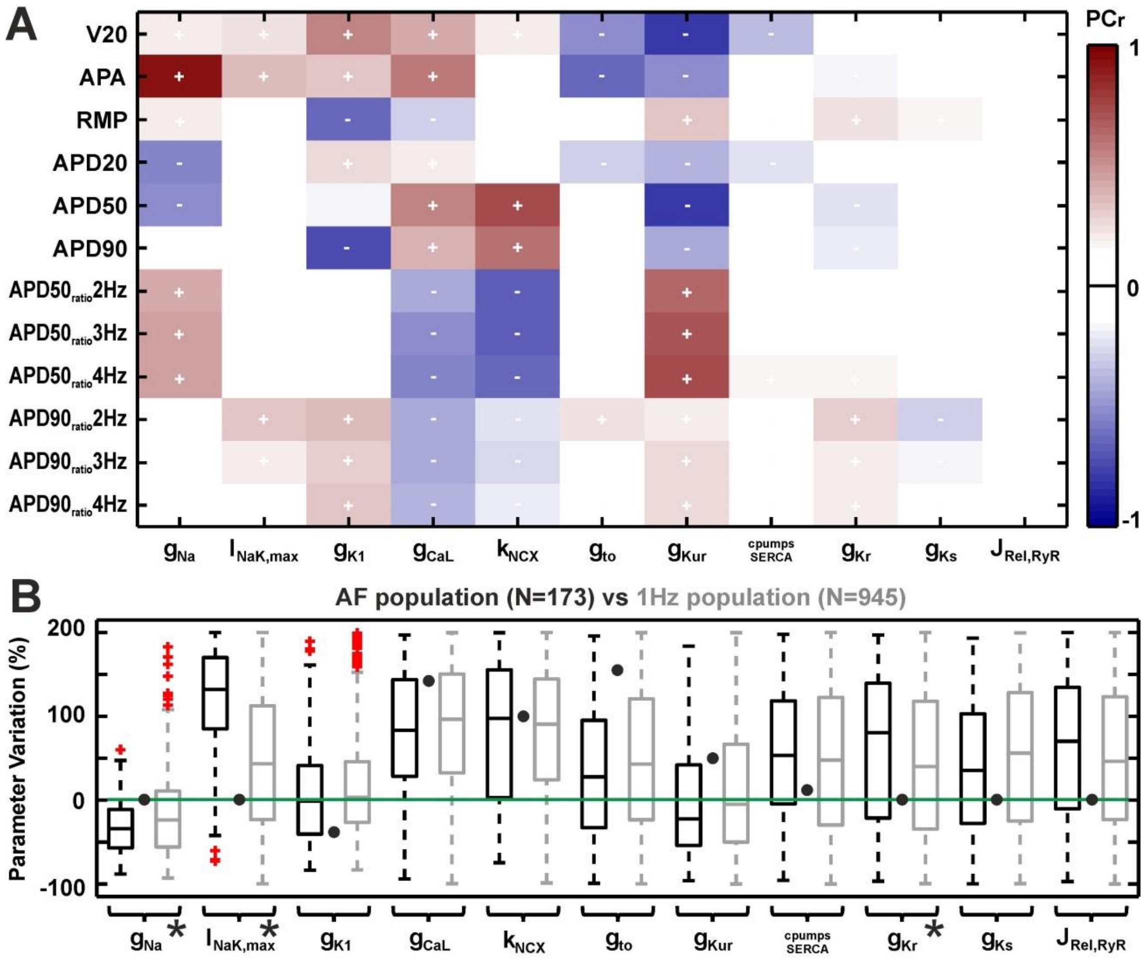

175
